# Baseline Quality of Life Factors Predict Long Term Survival after Elective Resection for Colorectal Cancer

**DOI:** 10.1155/2013/269510

**Published:** 2013-11-27

**Authors:** Abhiram Sharma, Leslie G. Walker, John R. T. Monson

**Affiliations:** ^1^Department of Colorectal Surgery, University Hospital of South Manchester, Manchester M23 9LT, UK; ^2^Institute of Rehabilitation, University of Hull, Hull, UK; ^3^Division of Colorectal Surgery, University of Rochester Medical Center, Rochester, NY, USA

## Abstract

*Background*. Studies have shown an association between baseline quality of life (Qol) and survival in advanced cancers. The aim of this study was to investigate their predictive value in long term survival after elective colorectal cancer resection. *Methods*. A consecutive series of patients undergoing elective colorectal cancer surgery for nonmetastatic disease were recruited in 2003/04. Patients completed standardized quality of life questionnaires (HADS, FACTC, MRS, and PANAS) prior to and 6 weeks after surgery. Univariate (log-rank test) and multivariate analyses (Cox proportional hazards) were performed to predict long term survival. *Results*. Ninety-seven patients met the inclusion criteria. Sixty-five (67%) were male and the median age of the group was 70 years. Forty-six (47.5%) patients had died and the mean survival was 1,741 days (median 2159, range 9–2923 days). Preoperative mood rating scale and functional assessment of cancer therapy-colorectal FACT C emotional well-being and postoperative FACT C additional concerns were independent predictors of long term survival. *Conclusion*. Incorporating psychosocial measures in preoperative assessment of cancer patients could help to identify patients who require assessment with a view to implementing psychosocial interventions. These active interventions to maximize mood and well-being should form an integral part of multidisciplinary treatment in these patients.

## 1. Introduction

Quality of life (Qol) is a critical aspect of living with cancer and there is increasing realization that beneficial and adverse changes in Qol are very important for patients and caregivers [1]. Qol measurements have become an integral part of cancer trials and the resultant available Qol data has shown a strong association between Qol and survival in cancer populations [2, 3]. Over the years, the quality of Qol data has also significantly improved allowing more meaningful analysis. Efficace et al. found a significant improvement in the quality and completeness of data and described over 60% of studies as being robust enough to guide clinical decision making [2, 4].

One of the earliest studies showing an association between quality of life and survival was published in 1987 and reported a significant relationship between changes in patient-rated well-being and survival in women receiving treatment for advanced breast cancer [5]. Similar results were published in 1991 in patients with metastatic lung cancer [6]. Numerous studies since then have reported the link between Qol and survival in varying cancers. Publications include predominantly lung and breast cancer [6–9] and also include head and neck malignancies [10] and renal cell cancer [11].

Most published studies demonstrating a significant association between Qol and survival have been in patients with breast or lung cancer. However, Maisey et al. pooled data from four randomized trials performed at their institution and showed that baseline Qol was a strong independent predictor of survival in patients with advanced colorectal cancer [12]. Another study demonstrated that social functioning was an independent predictor of survival in metastatic colorectal cancer [13].

Two recent systematic reviews have also found that quality of life factors predict survival in cancer patients [2, 3]. Gotay et al. conducted a systematic review of 39 studies (*n* = 13.874) and found patient reported outcomes to be significant predictors of survival in 36 studies. Quinten et al. examined 10, 108 patients from 30 trials including 1141 colorectal cancer patients. Overall survival in this cohort was 18 months. Age, sex, distant metastases, physical functioning, pain, and appetite loss were independent predictors of survival.

The majority of these studies, however, were carried out with patients who had advanced or metastatic cancer and who had a short survival time. This raises two issues. First, Qol may be poor due to the advanced nature of the disease and the realization by patients that prognosis is poor. Second, it is not clear if the findings can be generalized to patients with early stage disease with longer survival times. In fact, the three studies that did not show an association between Qol and survival were all conducted with nonmetastatic breast cancer patients (Gotay et al.). There are no studies exploring association between Qol and long term survival in nonmetastatic colorectal cancer.

The aim of this study, therefore, was to evaluate the effect of baseline Qol scores on long term survival in patients with nonmetastatic colorectal cancer undergoing elective curative resection.

## 2. Methods

### 2.1. Patient Recruitment

This was a prospective study of a consecutive cohort of patients with newly diagnosed nonmetastatic colorectal cancer scheduled for elective open resection in a single hospital trust in the UK. Exclusion criteria were Karnofsky performance status less than 80%, being unwilling or unable to give informed consent, or being unable to complete the study questionnaires.

Permission to carry out the study was obtained from the Local Research Ethics Committee (LREC/08/03/168, 11/09/2003). All patients completed informed consent. Patient recruitment was started in October 2003.

### 2.2. Time Points

#### 2.2.1. Baseline

Patients were recruited in the preassessment clinic 5–12 days prior to surgery. Patients gave written, informed consent and then completed various questionnaires (see Section 2.3).

#### 2.2.2. After Surgery

Patients attended a postoperative clinic between six and ten weeks after surgery (six weeks after discharge from hospital) and completed the same set of questionnaires.

#### 2.2.3. Clinical Followup

Patients were followed up according to a clinical protocol which includes visits every 3 months for the first year, visits every 6 months in the second year, and yearly visits up to at least 5 years. Data were recorded prospectively on a colorectal cancer database. Last followup for this study in October 2011 was done by electronic record search of every patient including a complete search of the death registry to determine overall survival. Survival status was recorded and the number of days from surgery to death was calculated as applicable.

### 2.3. Psychosocial Measurements

(1) The functional assessment of cancer therapy questionnaire with the colorectal module (FACT-C) was used to assess various aspects of quality of life. This questionnaire gives separate scores for physical well-being (FACTC PW), social and family well-being (FACTC SW), emotional well-being (FACTC EW), functional well-being (FACTC FW), and Qol relevant specifically to colorectal cancer (additional concerns) (FACTC AC). Validity and reliability have been reported [14].

(2) The hospital anxiety and depression scale (HADS) is a 14-item self report scale that was originally developed to screen for clinically significant anxiety and depression in the setting of a medical outpatient clinic [15]. The HADS is widely used in cancer research, and its validity and reliability have been reported in patients with cancer [16].

(3) The positive and negative affect schedule (PANAS) was developed by Watson et al. [17] as a brief measure of both negative affectivity (NA) and positive affectivity (PA). Several studies have confirmed the validity and reliability of PANAS and shown its relationship to other measures [18].

(4) The mood rating scale (MRS) was developed as a brief, acceptable, reliable, and valid measure of mood [19]. It consists of six visual analogue scales (150 mm) with verbally defined anchor points. The MRS has been shown to correlate well with the corresponding dimensions of the Profile of Mood States (POMS) and it has been shown to be sensitive to the effects of various interventions [20].

### 2.4. Statistical Analysis

All Qol scores were treated as categorical variables by performing a median split. Age was split into 65 and above and below 65. The Kaplan-Meier method was used for univariate analysis to estimate survival curves. The Cox proportional hazards regression model was used for multivariate survival analysis. All variables with a *P* value of <0.1 on univariate analysis were then entered simultaneously into a Cox proportional hazards model. Endpoint was overall survival, measured in days from date of surgery to death. Data were analyzed using the Statistical Package for the Social Sciences (IBM SPSS Statistics software, Version 20 (2011 SPSS, Inc.)).

## 3. Results

One hundred and seventeen patients undergoing elective colorectal resection were approached for participation; 103 agreed to participate in this study. Five patients died in the postoperative period before the second assessment. Fifty-one patients (52.5%) were alive at the final followup and the mean survival at last followup was 1741 days (median 2159, range 9–2923 days).

Sixty-five patients were men and the median age of the population was 70 years. The median Qol scores are listed in Table 1. The response rate varied for the various questionnaires and is listed in Table 1. Data was available for 85 to 97 patients.

TNM stage was a significant predictor of long term survival on univariate analysis (Table 2). Gender and age were not found to be statistically significant predictors.

PANAS positive affect (*P* = 0.05), mood rating scale (*P* = 0.04), FACT C EW (*P* = 0.05), and FACT C FW (*P* = 0.05) predicted long term survival on univariate analysis. Preoperative mood rating scale total score and FACT C emotional well-being were independently predictive of long term survival on multivariate analysis with TNM stage as a covariate. Better mood predicted better survival, whereas decreased emotional well-being predicted poorer survival (Tables 3 and 4).

Postoperative HADS anxiety (*P* = 0.02), HADS depression (*P* = 0.01), PANAS negative affect (*P* = 0.04), FACT C FW (*P* = 0.02), and FACT C AC (*P* = 0.01) were all statistically significant predictors of long term survival on univariate analysis. However, FACT C additional concern was the only postoperative Qol score independently predicting long term survival when TNM stage was used as a covariate (Tables 5 and 6): more concerns predicted poorer survival. TNM remained an independent predictor of survival in multivariate analysis.

## 4. Discussion

This study shows for the first time that baseline Qol measurements (MRS, FACT C EW, and AC) independently predict long term survival in nonmetastatic colorectal cancer patients. These Qol variables were significant univariate predictors of long term survival and retained significance in a multivariate model including TNM stage as a covariate. To our knowledge, this has not been reported before.

Patients self-reported social functioning has been shown to be an independent prognostic factor in metastatic colorectal cancer. Efficace et al. showed that there was a 6% increased risk of earlier death for every 10-point decrease in social functioning on the EORTC scale [21]. Global Qol score has also been shown by Maisey et al. to be an independent predictor of survival (*P* < 0.0001) in patients with advanced colorectal cancer. They recommended routine measurements in clinical trials to stratify cohorts and aid in trial comparison [12].

The study reported here is the first to include a cohort of patients with only nonmetastatic colorectal cancer. Perhaps most significantly this study followed up patients for a significantly longer time than other studies. Stage IV disease was excluded and median survival on followup exceeds 5 years. The key finding is that quality of life factors were significant predictors of survival in this group of patients with nonmetastatic colorectal cancer even after TNM stage was used as a covariate.

In their review of 30 studies, Quinten et al. highlight the issue of poor compliance with Qol assessment as a methodological difficulty and note that survival may be poorer in patients who did not complete the questionnaires. In the present study, high response rates—approximately 90% for the baseline and over 80% for postoperative questionnaires—were obtained. The high response rate is most likely due to the fact that preoperative and postoperative assessments were carried out by a member of the research team at a dedicated clinic. Patients were sent follow-up questionnaires by post 12 months after surgery and the response rate was significantly lower (35%). These data were therefore not used in the present study.

The association between aspects of Qol and survival is clearly consistent across several studies, but the reasons remain unclear. It is possible that Qol questionnaires measure the severity of disease more accurately or earlier than traditional measures. There is no direct evidence to corroborate this, although, in a study of patients with colorectal cancer with liver metastases, Qol was a more accurate predictor of survival compared to the number or volume of metastases [22]. It has also been suggested that higher Qol may be associated with positive behaviors like adherence to therapy or a healthy life style thus improving survival [23, 24].

There is also some evidence that Qol may influence tumor behavior to alter survival. This may be due to psychoneuroimmunological (PNI) effects on or of the tumour [20]; for example, various cancers have been shown to cause an increase in levels of proinflammatory cytokines: indeed cancer-related fatigue may be due to a rise in these cytokines in breast cancer patients [25]. Proinflammatory cytokines have also been shown to independently predict physical, cognitive, and emotional functioning in patients with advanced cancer [26]. Qol factors have been shown in our previous work to predict higher cytokine levels in cancer patients [27]. However, further studies are required to elucidate these possibilities.

The evidence for an association between Qol and survival is persuasive and this study adds further credence to the importance of measuring Qol. Qol measurements provide prognostic information beyond other traditional measures and changes in Qol may be an early marker of disease progression. These factors may also help to improve stratification in clinical trials and outcomes could be compared based on measurements of Qol. The implication that Qol levels may affect survival also carries the potential that improvement in Qol may improve survival in cancer patients. However, direct evidence to support this hypothesis is limited and inconsistent [28–30] and this should be an area for future research.

## 5. Limitations

The main limitation of this study is the relatively small number of patients recruited. As a result, median split of Qol scores was used rather than the described standardised cutoffs. We used a number of Qol variables and there is a risk of collinearity. However, multivariate analysis was used to counter this effect and some Qol factors were found to be significant independent factors. Despite these limitations, the study gives further justification for incorporating Qol instruments in the clinical management of cancer patients as well as in interventional research.

## 6. Conclusion

Poorer emotional well-being and greater additional concerns independently predict poor long term survival, while better mood predicts longer survival in nonmetastatic colorectal cancer. Incorporating these quality of life measures in preoperative and postoperative assessment of cancer patients would identify patients who require further assessment with a view to implementing a psychosocial and/or psychopharmacological intervention to alleviate distress. These active interventions to maximize mood/well-being should form an integral part of multidisciplinary treatment in these patients. In addition, the possible effects on survival of interventions in these distressed patients should be studied in larger cohorts.

## Figures and Tables

**Table 1:** Median scores of demographic and Qol measures.

	Available data	Median
Age	97	70 (39–86)
Gender	97	65 male (67%)
Preoperative HADS anxiety	97	6 (0–18)
Preoperative HADS depression	97	3 (0–16)
Preoperative PANAS positive affect	81	29 (14–50)
Preoperative PANAS negative affect	81	16 (10–40)
Preoperative mood rating scale (MRS)	94	524 (179–900)
Preoperative FACT C physical well-being	96	25 (7–28)
Preoperative FACT C social well-being	96	25 (5–28)
Preoperative FACT C emotional well-being	96	19 (5–24)
Preoperative FACT C functional well-being	96	22 (4–28)
Preoperative FACT C additional concerns	95	22 (8–28)
Postoperative HADS anxiety	85	5 (0–16)
Postoperative HADS depression	85	4 (0–15)
Postoperative PANAS positive affect	80	30 (11–50)
Postoperative PANAS negative affect	81	14 (10–41)
Postoperative mood rating scale (MRS)	84	542.5 (87–897)
Postoperative FACT C physical well-being	85	23 (8–28)
Postoperative FACT C social well-being	85	24 (8–28)
Postoperative FACT C emotional well-being	85	20 (4–24)
Postoperative FACT C functional well-being	85	16 (1–28)
Postoperative FACT C additional concerns	85	19 (7–28)

**Table 2:** Univariate analysis of demographic and staging factors and survival.

Factor	*P* value
Gender	0.27
Age	0.16
TNM stage	<0.001

**Table 3:** Univariate analysis of preoperative Qol and survival.

Qol measure	*P* value
HADS anxiety	0.38
HADS depression	0.78
PANAS positive affect	0.05
PANAS negative affect	0.63
Mood rating scale	0.04
FACT C PW	0.31
FACT C SW	0.21
FACT C EW	0.05
FACT C FW	0.05
FACT C AC	0.07

**Table 4:** Multivariate analysis of preoperative Qol and survival with TNM stage as covariate.

	Predicted change in hazard for one unit change in variable	Significance (*P*)
PANAS positive affect	0.60 (0.26–1.39)	0.23
Mood rating scale	0.29 (0.11–0.79)	0.01
FACT C EW	7.00 (2.57–19.04)	<0.001
FACT C AC	0.50 (0.23–1.11)	0.09
FACT C FW	0.60 (0.25–1.41)	0.24
TNM	3.68 (1.78–7.62)	<0.001

**Table 5:** Univariate analysis of postoperative Qol and survival.

Qol measure	*P* value
HADS anxiety	0.02
HADS depression	0.01
PANAS positive affect	0.70
PANAS negative affect	0.04
Mood rating scale	0.08
FACT C PW	0.15
FACT C SW	0.42
FACT C EW	0.16
FACT C FW	0.02
FACT C AC	0.01

**Table 6:** Multivariate analysis of postoperative Qol and survival with TNM stage as covariate.

	Predicted change in hazard for one unit change in variable	Significance (*P*)
HADS anxiety	1.012 (0.39–2.58)	0.98
HADS depression	0.82 (0.22–3.05)	0.76
PANAS negative affect	1.07 (0.33–3.47)	0.90
Mood rating scale	1.20 (0.48–3.0)	0.68
FACT C FW	0.56 (0.16–1.89)	0.81
FACT C AC	0.37 (0.16–0.89)	0.02
TNM	4.35 (1.93–9.8)	<0.001
